# A Comprehensive 6A Framework for Improving Patient Self-Management of Hypertension Using mHealth Services: Qualitative Thematic Analysis

**DOI:** 10.2196/25522

**Published:** 2021-06-21

**Authors:** Ting Song, Fang Liu, Ning Deng, Siyu Qian, Tingru Cui, Yingping Guan, Leonard Arnolda, Zhenyu Zhang, Ping Yu

**Affiliations:** 1 Centre for Digital Transformation, School of Computing and Information Technology Faculty of Engineering and Information Sciences University of Wollongong Wollongong Australia; 2 Illawarra Health and Medical Research Institute University of Wollongong Wollongong Australia; 3 Department of Health Examination General Hospital of Ningxia Medical University Yinchuan China; 4 Key Laboratory for Biomedical Engineering of Ministry of Education College of Biomedical Engineering and Instrument Science Zhejiang University Hangzhou China; 5 Drug and Alcohol Service Illawarra Shoalhaven Local Health District Wollongong Australia; 6 School of Computing and Information Systems Faculty of Engineering and Information Technology University of Melbourne Melbourne Australia

**Keywords:** patient experience, mHealth, mobile phone, mobile app, intervention, self-management, high blood pressure, chronic disease management, qualitative research

## Abstract

**Background:**

Hypertension affects over 15% of the world’s population and is a significant global public health and socioeconomic challenge. Mobile health (mHealth) services have been increasingly introduced to support hypertensive patients to improve their self-management behaviors, such as adherence to pharmacotherapy and lifestyle modifications.

**Objective:**

This study aims to explore patients’ perceptions of mHealth services and the mechanisms by which the services support them to self-manage their hypertension.

**Methods:**

A semistructured, in-depth interview study was conducted with 22 outpatients of the General Hospital of Ningxia Medical University from March to May 2019. In 2015, the hospital introduced an mHealth service to support community-dwelling outpatients with self-management of hypertension. Content analysis was conducted by following a grounded theory approach for inductive thematic extraction. Constant comparison and categorization classified the first-level codes with similar meanings into higher-level themes.

**Results:**

The patient-perceived mechanisms by which the mHealth service supported their self-management of hypertension were summarized as 6A: access, assessment, assistance, awareness, ability, and activation. With the portability of mobile phones and digitization of information, the mHealth service provided outpatients with easy access to assess their vital signs and self-management behaviors. The assessment results gave the patients real-time awareness of their health conditions and self-management performance, which activated their self-management behaviors. The mHealth service also gave outpatients access to assistance, which included health education and self-management reminders. Both types of assistance could also be activated by abnormal assessment results, that is, uncontrolled or deteriorating blood pressure values, discomfort symptoms, or not using the service for a long period. With its scalable use to handle any possible information and services, the mHealth service provided outpatients with educational materials to learn at their own pace. This led to an improvement in self-management awareness and ability, again activating their self-management behaviors. The patients would like to see further improvements in the service to provide more useful, personalized information and reliable services.

**Conclusions:**

The mHealth service extended the traditional hypertension care model beyond the hospital and clinician’s office. It provided outpatients with easy access to otherwise inaccessible hypertension management services. This led to process improvement for outpatients to access health assessment and health care assistance and improved their awareness and self-management ability, which activated their hypertension self-management behaviors. Future studies can apply the 6A framework to guide the design, implementation, and evaluation of mHealth services for outpatients to self-manage chronic conditions.

## Introduction

### Background

Hypertension is one of the principal modifiable risk factors for cardiovascular diseases, especially for stroke and chronic renal diseases [1]. Without a clear pathophysiology or cure, it remains a major global burden of morbidity and mortality [1]. Alarmingly, rising living standards and their derived unhealthy lifestyles are accelerating the prevalence of hypertension annually. In 2015, an estimated 1.13 billion people worldwide had hypertension, leading to 19% of global deaths [2,3]. Hypertension also imposes a substantial economic burden on society and health care systems. The annual medical cost of hypertension is estimated at US $370 billion worldwide, representing approximately 10% of the global health care expenditure [4]. Thus, hypertension control is a priority for improving population health and containing chronic disease burden.

Hypertension treatment includes both pharmacological and nonpharmacological interventions [5]. These interventions are only effective if a patient engages in long-term, even lifelong, self-management behaviors, which include adherence to pharmacotherapy and lifestyle modifications. Patients need to take antihypertensive medication as often as prescribed and regularly measure blood pressure (BP) to monitor its efficacy [2,6]. In addition, they need to follow a healthy lifestyle, such as maintaining a healthy diet, performing physical activities, avoiding tobacco and unhealthy alcohol use, and managing mental stress [2,7]. However, for most patients, adherence to self-management behaviors is challenging. Less than 50% of patients adhere to hypertension self-management 1 year after initiating pharmacotherapy [8]. Nonadherent behaviors include failure to initiate pharmacotherapy and lifestyle modifications, take antihypertensive drugs at the prescribed frequency, adhere to long-term treatment, and monitor efficacy regularly [9,10]. These challenges can be addressed by assistive technology innovations, such as mobile health (mHealth) services.

### mHealth Services

mHealth refers to the use of mobile devices to deliver medical or public health services [11]. As mHealth services break the constraints of time, space, cost, and capacity, they can provide patients with low-cost, affordable, ongoing support to manage their chronic conditions, such as hypertension, which requires long-term, lifestyle-related care plans and continuous monitoring in the home environment [12]. mHealth services have multiple modes, such as SMS text messages, mobile apps, and interactive voice responses [13]. With the proliferation of smartphones and the advancement of cellular networks, mHealth apps are increasingly available for patients to access health care services. In 2018, approximately 318,000 mHealth apps were available worldwide [14].

The effectiveness of mHealth services in assisting patients with self-management of hypertension has been widely studied. Empirical evidence suggests that mHealth services can better assist patients in controlling BP than the conventional face-to-face care model [15-20]. A review of 23 studies on the effectiveness of mHealth services found that 16 (69.6%) studies demonstrated a positive effect on medication adherence and healthy behavioral modification [21]. Lu et al [22] also found that mHealth apps can improve patient experience, especially in health information exchange, physician-patient communication, and short-term outcome improvement. Nevertheless, after assessing 186 hypertension-targeted apps in Apple and Google Play stores, Alessa et al [23] found that only 30 apps (16%) were likely to be effective in assisting hypertension self-management. The common feature of these 30 apps was that they all had 3 or more functionalities, including (but not limited to) self-monitoring, reminders and educational information or automatic feedback. There was little information about the theoretical basis of many apps, and there was no evidence about their effectiveness and usability. Song et al [24] also found that the ineffectiveness of the mHealth service might result from a lack of theoretical guidance for assisting behavioral modifications. Hallberg et al [25] interviewed 49 patients who used an interactive mobile phone–based system to self-manage hypertension. The patients appraised the system as a useful tool for self-reporting health conditions, measuring BP, retrieving self-reported data, and receiving motivational messages. These had led to improvements in lifestyle, health knowledge, and better engagement. However, as the participating patients were all active mobile app users, this study may be susceptible to positive bias. Conversely, Morrissey et al [26] questioned the sustainability of mHealth services despite positive patient engagement. To date, the mechanism for mHealth services to assist patients with self-management of hypertension is unclear.

### Study Context and Aim

To pilot trial an mHealth service to improve patient hypertension management and population health, a large-scope, international, tripartite, collaborative program was conducted between the University of Wollongong, Australia; Zhejiang University, China; and the General Hospital of Ningxia Medical University, China, through a formal research partnership. The mHealth service entitled *BP Assistant* was developed by the Biomedical Informatics Laboratory at Zhejiang University to support patients in self-managing hypertension in Ningxia, a province in China with a high prevalence of hypertension because of high salt intake [19]. It was implemented in the General Hospital of Ningxia Medical University, the only tertiary hospital in the province, in November 2015. The hospital has more than 3000 beds and supports outpatients with hypertension self-management. In March 2019, 2079 patients were enrolled in the mHealth management program. This study explored patients’ perceptions of the mHealth service and the service mechanisms to support them in self-managing hypertension.

## Methods

### Ethics Approval

This study was approved by the Human Research Ethics Committee of the General Hospital of Ningxia Medical University, China. The registration number is ID2018-325. Oral and written consent was obtained from all participants before the interviews, and their anonymity was preserved. All information and audio recordings of the participants were kept confidential.

### Study Design, Setting, and Participants

This study was conducted using semistructured, in-depth interviews from March to May 2019 in the Department of Cardiology at the General Hospital of Ningxia Medical University. The study population included outpatients with hypertension who were registered to receive the mHealth service provided by this department. All participants had participated in a previous clinical trial (registration number ChiCTR1900026437) that evaluated the effectiveness of the service.

### The mHealth Service

The mHealth service includes 2 technical components: a smartphone-based app, entitled BP Assistant, for patients to use to self-manage hypertension, and a web-based portal for health care providers to monitor and communicate with their patients. The app can be used in both iOS and Android systems. There are 6 key functional modules in the app: health education, health management plan, health checkup, health report, reminder service, and performance ranking (Textbox 1).

**Six key functional modules of the BP Assistant hypertension self-management app.**

**Health Education**

- To provide educational information about hypertension and hypertension management

**Health Management Plan**

- To provide a to-do list, which requires users to record and upload personal health data, including blood pressure and heart rate, medication type and dose, weight, diet, exercise, and uncomfortable symptoms

**Health Checkup**

- To provide feedback to tell users whether their blood pressure is abnormal or normal

**Health Report**

- To provide visual daily and monthly reports based on a statistical analysis of input data

**Reminder Service**

- To allow users to set up and receive tailored reminders for health management plans

**Performance Ranking**

- To score and rank each user’s performance according to their degree of app use in a ranking list

Health care providers can check all uploaded data from the web-based portal whenever needed. In particular, if an anomaly is detected, such as a sudden change in vital signs, long-term loss of contact, or patient-reported discomfort, the system will automatically send out a warning signal to alert the health care providers to follow up with the patient and provide treatment assistance or guidance.

### Semistructured Interview Questions

The interview guide was developed by the first author in English and then translated into Chinese. Content validity was evaluated by a panel of 10 bilingual experts, including 4 cardiovascular medical specialists, 4 health informatics experts, 1 certified health manager, and 1 information systems expert, before translating back into English. Afterward, the interview guide was pilot tested for its face validity by 3 hypertensive patients who had used the app. Feedback from these patients was taken to further refine the interview guide to improve the understandability and relevance of each question (Multimedia Appendix 1).

### Sampling and Recruitment

Participants were selected using purposive, snowball, and theoretical sampling until the data were saturated, that is, no new themes emerged [27-29]. We purposefully selected participants with varying sociodemographic characteristics to ensure diversity in age, gender, and type of mobile operating system being used. Theoretical sampling, which involves simultaneously collecting, coding, and analyzing data to determine whom to approach next to generate new insights, was used to enrich emerging categories and provide guidance for data collection to reduce selection bias.

Participants who (1) were aged 18 years or more, (2) had a diagnosis of essential hypertension (hypertension without identifiable causes) based on the Seventh Report of the Joint National Committee on Prevention, Detection, Evaluation, and Treatment of High Blood Pressure [5], and (3) had participated in the program to self-manage hypertension using the BP Assistant over 3 months were included in the study. Participants who (1) had secondary hypertension or (2) were unable to express their own perceptions because of mental disabilities or inability to speak were excluded from the study.

Potential participants were contacted by a registered nurse through a phone call or in a waiting room while they were waiting for a medical consultation. They were informed about the purpose and requirements of the study and then sought for verbal consent. An interview appointment was then made with those who provided verbal consent. At the beginning of the appointment, the interviewer described a detailed interview process and sought written consent. Only those who provided written informed consent were interviewed.

### Data Collection

After providing written informed consent, the participant was guided into a reception room where the interview was conducted. After briefing the participant on the purpose, procedure, and content of the interview, the interview was started. Each interview lasted approximately 30 minutes and concluded when information saturation was reached. The audio recordings were transcribed verbatim in Microsoft Word documents. All names included in the manuscript were replaced by codes to preserve the anonymity and privacy of the participants. All information and audio recordings of the participants were kept confidential.

### Data Analysis

The interview transcripts were systematically analyzed using an inductive thematic analysis method to identify different points of view and grouping them into various themes [30,31]. A total of 3 researchers engaged in content analysis. Each transcript was read carefully to obtain a general impression of the main experience of the participants by 2 researchers (TS and SQ). Content analysis followed the grounded theory approach [32,33]. The coding and classification were conducted in Microsoft Excel, benefiting from its affordance of easy visualization of a large number of codes in one screen, which was convenient for constant comparison, classification, and aggregation of codes, as suggested by Bree et al [34]. Each document was divided into sentences that only addressed one issue and were allocated to different cells in the same column. Each sentence was critically analyzed verbatim to identify the meaning unit by one researcher (TS), that is, an atomic text fragment containing relevant information to elucidate the research question. Given the different ways in which the participants expressed their experiences, each unit of experience was tagged with a label expressing its core meaning. This resulted in the abstraction of initial codes to describe these experiences.

The initial codes were compared, aggregated, and sorted into 16 higher conceptual–level concepts, known as subthemes. In this stage, how the patients were affected by the mHealth service was also established to understand the relationships. After that, concepts with similar meanings were further abstracted and grouped into an overarching 6 categories, called themes. After more than 3 months of constant comparison, aggregation, classification, and discussion, the tentative coding and data management were iteratively formulated and revised according to the consensus among the 4 researchers to avoid duplication and overlap (Table 1). The coding was then validated by another researcher (SQ), and the third researcher (PY) was referred to for arbitration when inconsistencies occurred.

**Table 1:** Examples of initial codes, subthemes, and themes from the interview.

Quotation	Initial code	Subtheme	Theme
“It is very convenient (when I am) outside (such as) going abroad. It can be taken and used everywhere.”	Convenient to use outside Convenient to use when going abroad Can be taken and used everywhere	Portability Portability Portability	Access Access Access
“Just won't forget it. I used to forget about (taking medication on time). Now once the alarm rings, I will take the medications.”	Do not forget Take medicine on time when the alarm rings	Reminder of self-management Cue to action	Assistance Activation
“I used to think hypertension was not a problem. (But) the cases (provided in the app) awaked me. It is my own business to manage my daily life.”	Not a problem Cases Own business	Importance of self-management Health education Importance of self-management	Awareness Assistance Awareness

## Results

### Demographics

A total of 22 outpatients with hypertension (4/22 women, 18%) participated in this study. Their ages ranged from 33-73 years, with a median age of 47 years. They were employed (11/22, 50%), self-employed (7/22, 32%), retired (3/22, 14%), or unemployed (1/22, 5%). Of the total participants, 41% (9/22) had a bachelor’s degree and above, 27% (6/22) had a college degree, 23% (5/22) held senior high school diplomas, and 9% (2/22) held junior high school diplomas. Out of 22 participants, 12 (55%) used iPhones, 8 (36%) used Android smartphones, and 2 (9%) used both.

### A 6A Framework That Explains the Mechanism for the mHealth Service to Assist Patient in Self-Management of Hypertension

#### Overview

**Figure 1:**
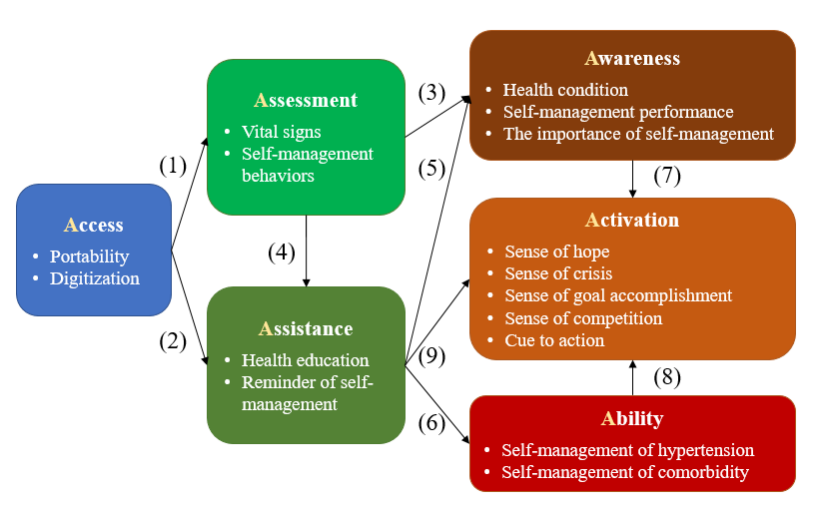
A 6A framework of using the mobile health service to assist patient in self-management of hypertension.

Patients’ perceptions of the mHealth service on their hypertension self-management are summarized in 6 themes (6A): access, assessment, assistance, awareness, ability, and activation (Figure 1).

With the portability of mobile phones and digitization of information, the mHealth service provided hypertensive outpatients with (1) low-threshold *access* to health *assessment* and (2) health care *assistance*. (3) The *assessment* results, that is, vital signs and self-management behaviors, gave the patients real-time *awareness* of health conditions and self-management performance. (4) In particular, abnormal results, such as deterioration of vital signs or lack of self-management behavior records, could also be fed back to health care providers to provide the patients with *assistance*. Health care assistance included health education and reminders of self-management behaviors. (5) Health education assisted the patients in improving their *awareness* of the importance of self-management and (6) the *ability*, that is, knowledge and skills, in performing self-management behaviors. The reminders assisted patients in overcoming the barrier of forgetfulness. The improvement of (7) self-management awareness, (8) ability, and (9) reminders together *activated* their self-management behaviors.

In descending order of the number of times mentioned by the interview participants, the 16 subthemes were vital sign assessment, health education, ability to self-manage hypertension, digitization, reminder of self-management, awareness of the health condition, cue to action, portability, self-management behavior assessment, awareness of the importance of self-management, sense of goal accomplishment, sense of hope, sense of crisis, awareness of self-management performance, sense of competition, and ability to self-manage comorbidities (Multimedia Appendix 2).

#### Access

The patients appreciated that mHealth lowered their threshold to access health information and health care services compared with the conventional hypertension care model. This was because of the portability of mobile phones and digitization of information.

##### Portability

The use of the mHealth service for hypertension self-management was available through the smartphones that patients carried with them in daily life. The lightweight, portable smartphones made it convenient for them to access health assessments and health care assistance without time and space limitations. The patients could keep their health care providers informed of their health conditions through access to the app. Remote communication and interaction reduced the need for hospital visits, avoiding excessive registration and examination costs:

It is very convenient [when I am] outside [such as] going abroad. It can be taken and used everywhere.

Patient 09

It’s easier for communication about hypertension management [with my doctor] because [if] there is always some tool to connect between us to exchange information, [I] do not have to run to the hospital. It has always been annoying to go to the hospital to seek medical treatment. We have to wait in a crowded space for a long time, and the queue is often quite long. Health problems that could be solved in a few minutes always took a whole morning.

Patient 18

##### Digitization

The mHealth service provided patients with a convenient digital platform to access health information and health care services. The data were in digital form and stored in the app, which was easier to retrieve and less likely to be lost than the traditional method of recording data in a notebook. It was also quicker and easier to review the records without flicking through paper-based records, allowing both patients and their health care providers to capture useful information in time:

In every record, the specific time was recorded; the specific data was also recorded...unlike writing it down in a notebook, it won’t be lost.

Patient 06

It’s so convenient that I can track my records easily,...,unlike before, if I needed to identify a certain record, I had to go through all written records one by one.

Patient 14

(It is) more convenient. I used to record my BP in a notebook, every time the doctor had to flip through it and read the records one by one. You see, it is impossible for her to read each record and identify my highest systolic BP. As she has to see so many patients in a day, in fact, she cannot get anything (meaningful)...(But) now she only needs to glance at the curve, the overall trend, the peaks and valleys directly on the mobile phone. This is faster and more intuitive.

Patient 02

#### Assessment

The patients praised the mHealth service because it provided them with the opportunity to assess their vital signs and self-management behavior.

##### Vital Signs

The mHealth service assessed the vital signs of patients, that is, BP and heart rate, measured and entered by themselves on the app through automatic analysis, with the results presented back to both the patients and their health care providers. The patients believed that these results provided evidence that enabled their health care providers to understand their condition, make an appropriate diagnosis, and provide feedback, such as adjusting the treatment program, instead of relying on a single measurement of BP in the clinic:

[The app] helped me to tweak my medicine well,...My medication had been changed four times before because my BP reading was always up and down. I did not have any records at that time...With the app, the doctor prescribed the medicine in reference to all the records of my BP. Once the medication was right, the BP was gradually under control.

Patient 11

One patient specifically pointed out that the mHealth service helped to solve his *white-coat* effect in the clinic:

...my BP readings are always higher when I sit with the doctor but lower at home. So, the data I upload [to the app] is more accurate than that taken in the hospital.

Patient 09

##### Self-Management Behavior

The mHealth service automatically recorded the patients’ self-management behaviors, including BP, heart rate, and weight measurement; medicine intake; step count; and food intake, to assess their self-management performance. The patients thought that the assessment results provided their health care providers with evidence to determine the reason for the suboptimal efficacy, that is, whether the medicine was inappropriate or the patients did not adhere to the self-management:

In the past, if the BP was not well controlled, the doctors would ask me if I take medicine on time or if there was too much salt intake or I did not do exercise. With the record, she can immediately see what might cause poor BP control.

Patient 17

In particular, abnormal assessment results, that is, uncontrolled or deteriorating BP values, discomfort symptoms, or not using the system for a long period could also be detected by the mHealth system. Once an abnormal result was detected, the mHealth system would send an alarm signal to notify the health care provider to contact the patient or their families to provide timely feedback:

...[The health manager] called me in time and asked me what happened recently; why wasn’t my BP controlled but suddenly increased? Did I eat high cholesterol food etc...[it] is a wake-up call to us.

Patient 07

The negative experiences reported by the patients included overwhelming assessment items, inaccurate assessment of health conditions, and inability to see how lifestyle changes impact BP levels. Some patients complained that the app required too many data items to be entered on a daily basis; they doubted about the actual effect on disease management but saw it as a waste of their time:

Some functions are useless. For example, I take the same drugs every day. Why do I need to upload them every day?

Patient 12

I only use [hypertension-]related functions. Also, it’s hard to assess, i.e., the diet. For example, if I record a bowl of rice, you still don’t know exactly how much rice I eat.

Patient 07

Some patients considered that some wordings in the app, such as BP and discomfort symptoms, were not entirely accurate:

Your assessment of discomfort symptoms is inaccurate and illogical. The symptoms listed in the app, such as chest pain and mixed-up words, are typical symptoms of cardio or cerebral infarction. How can I still stay at home and wait for feedback once they occur? I must have to directly go to the hospital or call the emergency. If you say sweating, a little dizzy, these can be called discomfort symptoms.

Patient 02

The assessment of BP is unscientific without considering the pressure difference. I remember one time, I felt particularly uncomfortable, and my BP was 100/80 [mmHg]. The app showed unexpectedly that my BP was normal. This would mislead the patient.

Patient 12

One patient strongly felt a lack of logical connection between the assessment results, for example, BP values and other data items. This impeded a patient’s recognition of the importance of certain app functions for hypertension management, thus reducing their acceptance and use of these functions:

The assessment indicators [in the app] are all isolated. We can’t see any connection between what we do and the corresponding outcomes.

Patient 09

#### Assistance

The patients reported 2 types of assistance provided by the mHealth service: health education and reminders.

##### Health Education

The patients acknowledged that their physicians had explained, more or less, the reason and the way to self-manage hypertension; however, they either did not fully understand or ignored these instructions during the medical consultation period. The mHealth service assisted them with this challenge because it provided a large volume of educational materials that were not otherwise accessible, which allowed them to learn at their own pace so as to absorb the information and to truly understand the meaning of the medical orders:

Yes, [the doctor] told me before. But, he or she may not go into the level of detail due to time limit. (I) didn’t take it to heart either [because I’ only paid attention to what medications were (at the time of medical consultation).

Patient 18

...the doctor had told me everything [about self-management of hypertension] when I was sitting with her. But I had little knowledge and did not really absorb the information. When encountering the same information again [in the app], I came to understand why and how to [do it].

Patient 03

The patients also felt that the content provided by the mHealth service was more trustworthy than those obtained from other sources because it was disease-focused and recommended by health care professionals in a prestigious tertiary hospital:

...although it is much easier to get the [hypertension-related] information from other sources, such as TV, mobile phone, newspapers, your app is more trustworthy because it is targeted, specifically for hypertension and it’s sophisticated. Also, [it is] professional because [the content is] checked by doctors, unlike information [from other sources which was] just copied and pasted [from somewhere else]. [They] cannot be trusted.

Patient 02

##### Reminders

The mHealth service reminded them to perform necessary self-management actions, such as taking medications, which assisted them in overcoming the barrier of forgetfulness:

Just won’t forget it. I used to forget about [taking medication on time]. Now once the alarm rings, I will take the medications.

Patient 09

The negative experiences mentioned included that the feedback service was lagged behind and untimely because they had experienced symptoms and discomfort without receiving any feedback:

...I felt chest pain at one time, and I reported it in the app. But there was no response.

Patient 22

There were also complaints about the technical instability of the app, preventing the patient from getting timely assistance:

Sometimes I can’t log into my account. You know, users will lose confidence in your product in this case.

Patient 15

Occasionally, it may fall offline.

Patient 13

#### Awareness

The patients felt that their awareness was improved by using the mHealth service, which included awareness of their health conditions and self-management performance and the importance of self-management.

##### Health Conditions and Self-Management Performance

The visual and colorful graphic assessment results charts for vital signs and self-management behaviors improved patients’ awareness of their health conditions and self-management performance, respectively:

...then translate [the assessment results] to a graph. I was more aware [about my health condition] as soon as I looked at the chart. My BP, for example, showed a downward trend but had not met the target.

Patient 02

It has several indicators, in different colors, it’s very vivid, clear at a glance.

Patient 05

##### Importance of Self-Management

Despite being diagnosed with hypertension, some patients never took it seriously because they did not believe in the consequences of the disease or the need to control it. The patients admitted that the educational materials assisted them with a better understanding of hypertension-related risk factors and corresponding consequences and built their belief in the responsibility of self-management. This made them change their attitudes, being aware of the importance of self-management, and they thus had the intention to engage in self-management behaviors:

When diagnosed with hypertension after a medical examination, I thought the doctor just scared me because I didn’t feel anything wrong with my health. I ate and drank and smoked whatever and whenever I wanted, and even didn’t take any medication because I was afraid of side-effect. After reading the materials in the app, I knew I was wrong. It was time to manage hypertension.

Patient 19

I have never managed [hypertension] before [using the app because] I thought many of my family and friends also had [it], but they were still alive and well. Now I know that once there are symptoms, it is late because our organs have been injured, and the injury was mostly irreversible.

Patient 04

I used to think hypertension was not a problem. If [I felt] dizzy, [then I] go to see a doctor; otherwise, just let it be. [But] the cases [provided in the app] awaked me. It is my own business to manage my daily life.

Patient 18

#### Ability

Patients reported that using the mHealth service improved their ability to self-manage hypertension, including their knowledge and skills in hypertension self-management and inspiration for self-management of comorbidities.

##### Knowledge and Skills in Self-Management of Hypertension

The health education provided by the mHealth service increased patients’ general knowledge of hypertension self-management and improved their BP control skills and strategies for practicing healthy lifestyles:

The health tips are quite useful, [I] got to know what should [I] pay attention to in the daily life, and how to achieve self-regulation; for example, I often remind myself not to be angry, to maintain a healthy diet, and to do physical activities.

Patient 15

...I went through the educational material little by little, such as how to measure BP correctly at home, what matters when taking antihypertensive drugs, etc. Now, I know how to self-manage [hypertension] well.

Patient 01

In particular, one patient mentioned that the mHealth service improved his ability to deal with discomfort symptoms. As he learned about the signs and patterns of the manifested symptoms and the level of severity, he was confident and capable of deciding whether to wait and see or to seek medical assistance immediately:

...The educational materials are quite useful...[For example, previously] I always rushed to the emergency department when I felt dizzy or heart palpitations because I had no idea what happened. But now what I usually do is taking out a gauge to measure my BP and heart rate, if they are abnormal, I will observe it for a while. Usually, they would return to normal without doing anything. Otherwise, I would see the doctor. In addition, I used to be very anxious when this case happened, which made my BP higher; but now, I always remind myself to be calm, making a rational decision after observation.

Patient 05

##### Inspiration for Self-Management of Comorbidities

The mHealth service also improved patients’ ability to self-manage comorbidities because there were similar requirements for managing chronic diseases:

Although this [app] is hypertension-focused, it is virtually enlightening to my daily self-management of coronary heart disease and diabetes, such as eating, exercising, medicine adherence, because their management principles are similar and interlinked.

Patient 09

However, some patients believed that the information provided by the app was not sufficient to improve their ability because the content was monotonous and difficult to absorb and the update was not frequent enough:

The content of health education is monotonous, and lack regular updates.

Patient 07

The health education material was not adequate, thus less useful. The information was out of date.

Patient 14

#### Activation

The patients reported that using the mHealth service activated their self-management behaviors, which included their sense of hope, sense of crisis, sense of goal accomplishment and sense of competition, and cues to action.

##### Sense of Hope and Crisis

The assessment results of vital signs gave the patients real-time awareness of their health conditions, thereby activating their sense of hope or crisis. Awareness of improved health conditions provided a sense of hope for BP control, which encouraged them to maintain self-management behaviors:

It brought up a sense of achievement by seeing the curve of my BP recording being flattened. This gives me the motivation to keep going. You see, [my systolic BP] has dropped from 164 [mmHg] to 142 [mmHg]. It will be normal once [it] drops to 120 [mmHg].

Patient 05

Conversely, awareness of deteriorating health conditions provided a sense of crisis, which activated them to improve self-management strategies:

A curve shown in the app can indicate whether my heart rate is fast, slow or normal today, like my BP. If abnormal, I was set on alert to reflect upon my own behavior. Should I get more exercise? Am I eating too much salt? Did I forget to take my medicine?

Patient 14

...if you don’t record [the BP], there is no [data] for comparison...But if you have recorded and compared yesterday’s, today’s and tomorrow’s records, a sense of crisis will push you to reflect. [For example], my BP is rising. What might have caused it? Oh, probably because I drank alcohol. The record showed my BP was normal before. So I shouldn’t drink alcohol in a couple of days; instead, I should pay attention to a healthy diet, regular work and rest.

Patient 15

##### Sense of Goal Accomplishment and Competition

The assessment results of self-management behaviors made the patients aware of self-management performance of both themselves and other users, thereby activating their sense of goal accomplishment and competition. Persistent recording of patients’ self-management efforts had brought them a sense of goal accomplishment:

...it would be nice that my effort could be faithfully recorded by your app. I did it without any recording in the past. But now, look at these records. I have the strength [to keep going].

Patient 15

What I did and not have been recorded [in the app]. For example, walking, I was looking through [the records] now and then. [If] I walked 9,000 steps yesterday, I knew I reached the standard and felt at ease. If I did not reach 9000 steps today, I always worried and thought that I should go out and walk more.

Patient 21

In particular, 2 patients mentioned that the performance-ranking function enabled activation. When a patient knew that other patients did better than they had done, their sense of competition was switched on, which activated self-management behaviors:

Seeing that someone was ahead of me, I made a firm decision that I had to catch up with him or her.

Patient 16

##### Cues to Action

The reminder activated patients’ self-management behaviors by providing them with prompts to perform a specific action, for example, taking medications:

...[the app] always reminds me to take medicine on time.

Patient 18

[Previously,] the doctor couldn’t track my health conditions, and I wasn’t completely sure [about it]...so I was anxious. But now, I was immediately told that I should go to the hospital and take a look at it, which made me felt safe. I felt that I was put on the radar screen. They had been paying close attention to me with timely feedback, so I should also play an active role to manage myself well.

Patient 03

Despite the perceived benefits of self-management activation, certain patients suggested further improvement in the incentivizing mechanism for using mHealth services:

Actually, [if] you want to encourage them, you have to send a message to list their achievement, such as how many tasks have been completed, how well they controlled [their BP], and how long they have persisted in using the app, etc.

Patient 07

...what can I get? For example, I use another app, and if I walk up to 5,000 steps, it will pop up a message saying, “Congratulations! You have reached 5,000 steps today. Please keep it up”. I can’t see recognition of what I achieved using this app.

Patient 09

I don’t know the criteria for scoring and ranking the performance, so I’m not motivated because I don’t know what to do next.

Patient 15

## Discussion

### Principal Findings

**Figure 2:**
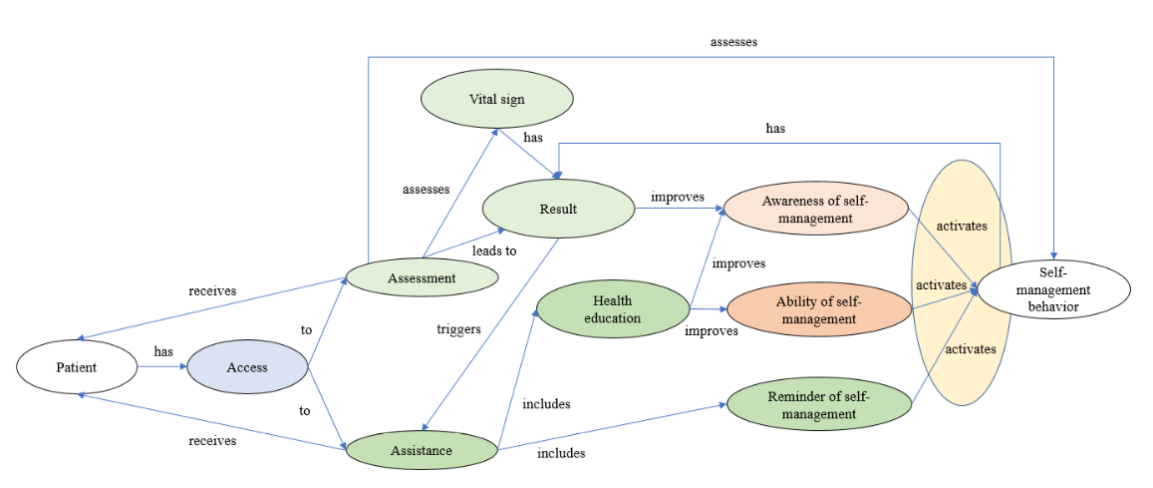
The mechanism for the mobile health service to support patients to self-manage hypertension.

Compared with the traditional hypertension care model, the mechanism for the mHealth service to support patient self-management of hypertension can be summarized in a 6A framework: access, assessment, assistance, awareness, activation, and ability (Figure 2).

**Figure 3:**
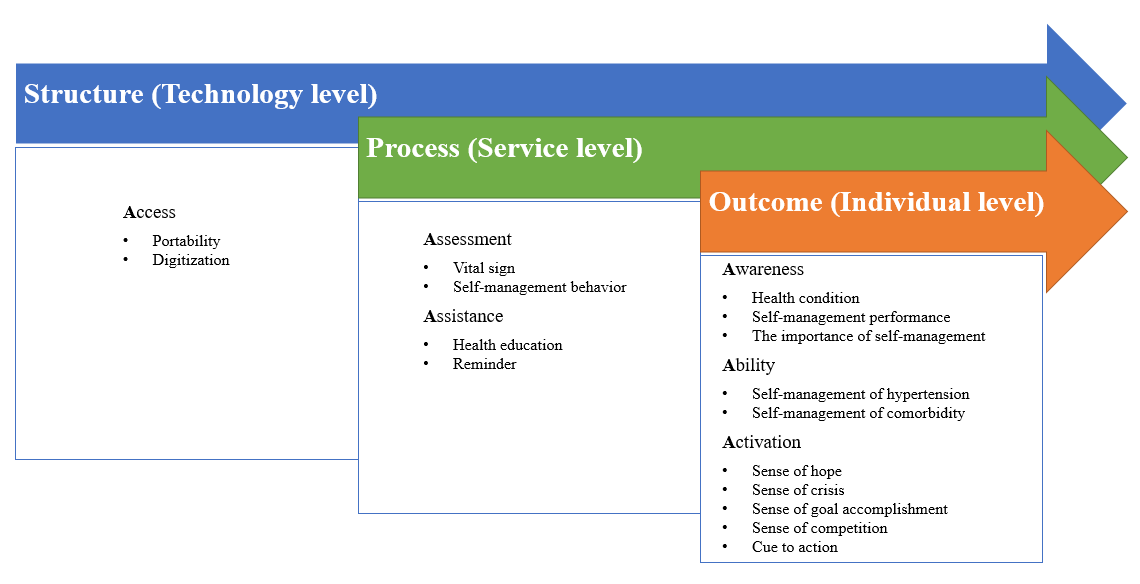
Interactions among the structure (access), process (assessment and assistance), and outcome (awareness, ability, and activation) in the mobile health service for hypertension self-management.

Our findings can be explained by Donabedian health care quality model [35]. The mHealth service changes the structure, that is, the delivery mode and process of the traditional hypertension care model. It provides patients with easy access to health assessment and health care assistance, which assists with improved self-management awareness and ability. These again activate self-management behaviors (Figure 3).

The mHealth service enables outpatients to access health care services that were previously only available to inpatients. These included the assessment results of vital signs and self-management behaviors and assistance from the mHealth system and health care providers. The increased access is attributed not only to the functions of the app but also to the changes in access rights facilitated by the mHealth delivery mode. Computer automation reduces labor costs to facilitate structural changes in the scope of health care service delivery beyond the boundaries of hospitals. This change in the health care service structure appears to lead to improved clinical processes [24]. Our patients perceive app use, or smartphone use, as an effective and easy way of gaining access to health care services, facilitating their acceptance and use of mHealth apps, as found by Anderson et al [36].

Smartphone portability encourages patients to interact with the app and extensively use app functions without geographical boundaries and time restrictions [37,38]. The patients also appreciate that the mHealth service lowers the threshold for them to access health care services. They do not need to make a long journey and wait in a long queue to see their health care providers, saving them time and cost.

The mHealth service also improves data access through timely data transfer and easier data retrieval [39-41]. Vital sign data are presented in easy visual graphics, allowing patients to read the information on a mobile phone at a glance, instead of reading it on paper or a computer. As suggested by Hallberg et al [25], if the graph can be viewed directly on a mobile phone without connecting to a computer, the mHealth service can be more useful because it addresses the barrier to using the system.

A total of 2 types of processes are changed—assessment and assistance. Patients have access to assessing their vital signs and self-management behaviors and the assistance of health education and reminders at home.

The assessment results improve patients’ self-awareness of their health conditions, that is, *What is my current situation*? It also provides evidence to health care providers for accurate and timely diagnosis and prescription, as found by Myers et al [42]. However, Den Hond et al [43] stressed that self-measurement of BP cannot replace ambulatory monitoring for clinical diagnosis. Interestingly, the *white-coat effect*, that is, rising BP when seeing a doctor, can distort the real health condition of the patients and lead to misdiagnosis and treatment in face-to-face consultations [44]. Regularly measuring BP at home and recording the results on the app can help solve this problem. This is in line with the findings of Barsky et al [45]. It is worth noting that some patients reported low accuracy of BP assessment results. If there is a false assessment result, it will inevitably affect the judgment of the severity of the disease and even lead to wrong actions. This indicates that future product development should involve medical experts to ensure assessment accuracy [46].

As suggested by Norman Cousins, “Each patient carries his own doctor inside him” [47]; self-management is essential for all patients with chronic conditions, such as hypertension. The first step was to establish awareness and ability. Lack of awareness of the importance of self-management is a major factor causing poor BP control [48]. Bokhour et al [49] also suggested that hypertensive patients’ awareness of factors that affect BP in their daily life may influence their ability to perform self-management behaviors. Our findings suggest that health education has improved patients’ awareness of health self-management by addressing their *why* questions, that is, *Why is it important to self-manage hypertension?* Despite best efforts, limited medical resources and time make it difficult to educate patients and meet their individual needs in traditional face-to-face interventions [50]. The advantage of the mHealth service comes from its high scalability for providing services, for example, disseminating a large amount of educational material to a wide range of patients with hypertension for effective health education for chronic disease management [51-53]. It also allows these patients to absorb the information in their own time and at their own pace until they are fully aware of the importance and have the ability to perform self-management. They can also continuously improve their self-management abilities through constant learning. This is in accordance with the basic requirements for successful pharmacotherapy and lifestyle management for hypertension. However, some patients were disappointed with the publication frequency, accuracy, and legibility of the educational content. This suggests that a successful mHealth service requires accurate resources to be published regularly to sustain patients’ trust with the mHealth service, which was in accordance with the study by Zarea et al [54] to evaluate health information websites. The negative feedback from the patients also provided insights into what and why the mHealth services did not work, that is, violating the 6A mechanism.

Awareness of one’s health conditions and self-management performance can activate a person to perform self-management behaviors [55]. This is likely to be achieved by stimulating their sense of hope, sense of crisis, sense of goal accomplishment, and sense of competition, which are the internal driving forces for behaviors. Deteriorating BP changes can enable patients to take responsibility for managing their own health, activating the urge to perform self-management behaviors. This can be explained by the protection motivation theory, which states that protective motivation is affected by the perceived severity of the disease [56]. A similar phenomenon was found in patient self-management of chronic kidney disease, chronic fatigue syndrome, and unhealthy alcohol use [57-59].

Forgetting to perform self-management is a common problem facing patients with hypertension in daily life. The automatic reminder provides a prompt in solving this problem. It serves as an external driving force to complement the internal driving forces mentioned above. This is in line with the health belief model, suggesting that a *cue* is essential to guide people to perform health-promoting behaviors, especially for the older people [13,24,46,60].

Our results also corroborate and complement the existing behavior change theories, such as the COM-B (Capability, Opportunity, Motivation, Behavior) model proposed by Michie [61]. The Capability, Opportunity, Motivation, Behavior model describes the interactions between 3 components—capability (C), opportunity (O), and motivation (M)—which produce behaviors (B) that, in turn, affect these components. *Capability* is defined as the individual’s mental and physical capacity to engage in related activities. It is similar to *ability* in our 6A framework, that is, the patient’s knowledge and skills to perform self-management. *Opportunity* is defined as all external factors that make the behavior possible or prompt it. It corresponds to access, assessment, and assistance provided by the mHealth service in the 6A framework. *Motivation* is defined as all brain processes that can stimulate and guide behavior, similar to *activation* in the 6A framework, which includes habitual processes (cue to action) and emotional responses (sense of hope or sense of crisis or sense of accomplishment or sense of competition). One step further, the 6A framework highlights the concept of *awareness*. This is supported by another behavioral change theory, the Theory of Planned Behavior. According to the Theory of Planned Behavior, the premise of a behavior change is the intention to change [62]. In contrast, internal capabilities and external opportunities are not adequate conditions for behavioral change. In the patients’ stories, it was repeatedly mentioned that the driving force for them to engage in mHealth service is *awareness* of the need to change and the mechanisms to make change. Therefore, improving self-*awareness* is one of the key mechanisms for mHealth services to function.

### Limitations

Our findings were drawn from interviews of 22 patients to understand their subjective perceptions of the mHealth service. Therefore, the findings were influenced by their own experience, despite the fact that studying patients’ perceptions is a rational approach for understanding information system performance [39]. Confinement of the study to a single hospital may limit the generalizability of the results to other settings. There is a limitation in translating the results of a mobile app intervention for hypertension management to other mHealth services media, such as text messaging and interactive voice response [13,59], or for other chronic diseases, such as diabetes. Different apps can have different functions and delivery formats. This suggests the need for further studies on patients’ needs and preferences to understand which functions and mode of delivery are most helpful to them. Moreover, the analysis can be difficult to be completely free from a priori views and knowledge of the hypertension management system of the authors, an inevitable limitation of qualitative content analysis [33]. However, each sentence in the transcripts was read verbatim to understand the meaning of each participant as neutral and accurately as possible. The verification of the analysis by 2 more researchers also made up for this limitation as much as possible.

### Conclusions

The mHealth service extended the structure of the traditional hypertension care model beyond the hospital and clinician’s office. With its portability and digitization, the mHealth service provided patients with low-threshold access to communicating with their health care providers and receiving health care services to support their self-management of hypertension at home. Such structural changes in health service delivery have brought process improvement to assist patients’ access to effective health assessment and health care assistance anytime, anywhere. The improvement in awareness and self-management ability and reminders brought about by such structural and process changes activated their hypertension self-management behaviors. They would like to see the mHealth service to provide more useful functions and easy-to-use services. Therefore, the comprehensive 6A framework extracted from the in-depth qualitative research theorized the mechanism for the mHealth service to improve patient self-management of hypertension. The mechanism can be further applied to guide the design, implementation, and evaluation of mHealth services for outpatients to self-manage chronic conditions.

## Appendix

Multimedia Appendix 1 Interview guide.

Multimedia Appendix 2 The number of times each subtheme was mentioned by the participants in the interviews.

## Abbreviations

BP: blood pressure
COM-B: Capability, Opportunity, Motivation, Behavior
mHealth: mobile health
