# Supplementary material for: A Comprehensive 6A Framework for Improving Patient Self-Management of Hypertension Using mHealth Services: Qualitative Thematic Analysis
Source: J Med Internet Res. 2021 Jun 21;23(6):e25522. doi: 10.2196/25522 (PMC8277389; doi:10.2196/25522)
Supplement: Multimedia Appendix 1 [file jmir_v23i6e25522_app1.pdf]

Appendix 1. Interview guide

1. What was your initial reaction when you were recommended to use “Blood Pressure Assistant”?
2. Do you like to use it? Why or why not?
3. When was your hypertension diagnosed, and how was it managed before using the app “Blood Pressure Assistant”?
4. What do you know about hypertension before using the app “Blood Pressure Assistant”? Where did you acquire the information?
5. How long have you used the app “Blood Pressure Assistant” to manage your hypertension?
6. What did you usually use the app for? (Which functions did you use? Provide a whole list of the functions of the app for the participant to recognise) Do you find this app useful? Which functions are more useful? Why?
7. Which functions that you did not like to use? Why?
8. Do you have concerns or difficulties in using this app in your everyday life?
9. What improvements would you like to see?
10. What is your role in managing hypertension? What is your healthcare providers’ role? What is the role of the app?
11. Are there any further issues that you would like to discuss with me?
