# Supplementary figures and images for: A Comprehensive 6A Framework for Improving Patient Self-Management of Hypertension Using mHealth Services: Qualitative Thematic Analysis
Source: J Med Internet Res. 2021 Jun 21;23(6):e25522. doi: 10.2196/25522 (PMC8277389; doi:10.2196/25522)

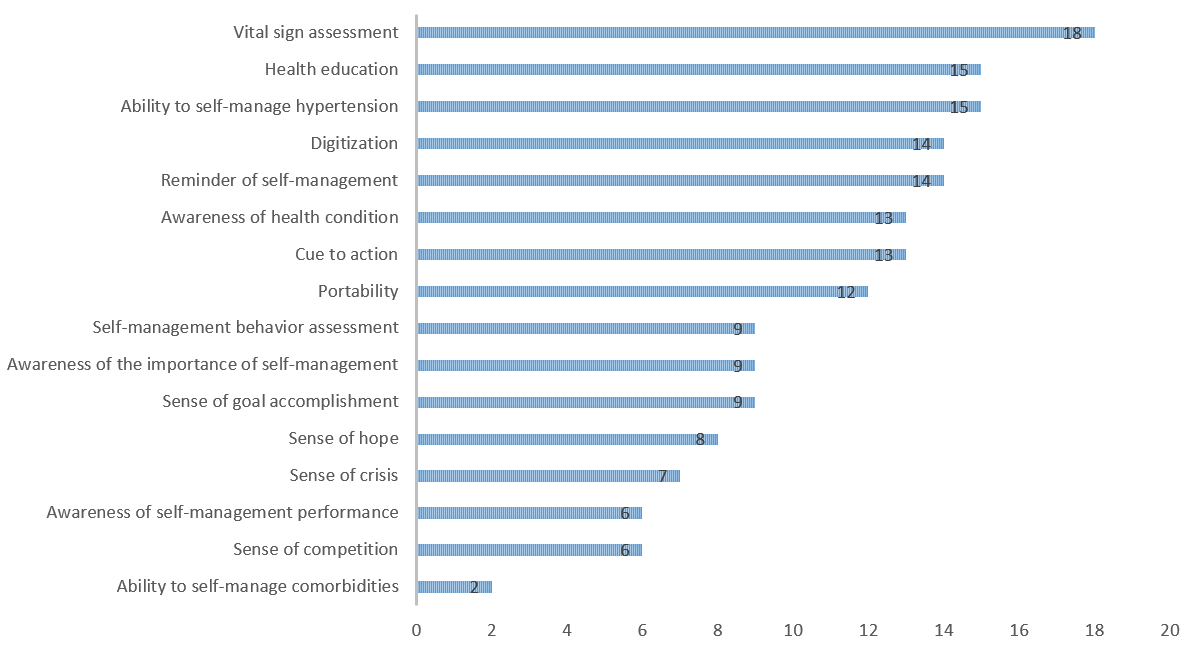

Supplement: Multimedia Appendix 2 [file jmir_v23i6e25522_app2.png]
